# The Transactions of the New Hampshire Medical Society, (Sixty-Eighth Anniversary)

**Published:** 1856-11

**Authors:** 


*The Transactions of the New Hampshire Medical Society, (sixty-eighth anniversary.)* Held at Concord, June 3d and 4th, 1856.

We are indebted to Dr. Geo. H. Hubbard, for our copy of these Transactions. They contain an able annual address by the president, Dr. Adoniram Smalley, on "Progressive Medicine: its relations to society, the other sciences and professions;" a very interesting paper by Prof. Edward H. Parker, entitled "Results of the Quantitative and Qualitative Analysis of Homœopathic Medical Preparations," which we publish in our present No.; a Report on Quackery, by Dr. Albert Smith, in which the high ground of non-intercourse is recommended; a Report on Practical Medicine, by Dr. Wm. H. H. Mason, a matter-of-fact sort of paper; a Report on Indigenous Botany, by Dr. Albert Smith; a dissertation on Inflammation, by Dr. P. A. Stackpole; and finally, the Report of the Delegates sent to attend the examination of Dartmouth College. Altogether the present volume is an interesting one, characterized rather by sound sense than brilliancy.

